# Feasibility and User Experience of Immersive Virtual Reality–Based Rehabilitation in Patients With Stroke: Single-Arm Pretest-Posttest Pilot Study

**DOI:** 10.2196/79584

**Published:** 2026-03-11

**Authors:** Sara Arlati, Marta Mondellini, Isabella Martinelli, Eleonora Guanziroli, Mauro Rossini, Marco Sacco, Franco Molteni

**Affiliations:** 1 Institute of Intelligent Industrial Technologies and Systems for Advanced Manufacturing, National Research Council, via Previati 1/E, Lecco, 23900, Italy, 39 034123 ext 50202; 2 Department of Psychology, Università Cattolica del Sacro Cuore, Milano, Italy; 3 Institute of Health, School of Health Sciences, HES-SO Valais-Wallis, Sion, Switzerland; 4 Department of Brain and Behavioral Sciences, University of Pavia, Pavia, Italy; 5 Villa Beretta Rehabilitation Center, Ospedale Valduce, Costa Masnaga, Italy; 6 Ospedale Valduce, Como, Italy

**Keywords:** motor training, user experience, subjective assessment, chronic patients, neuroplasticity

## Abstract

**Background:**

Immersive virtual reality (VR) is promising in stroke rehabilitation; it is believed to promote motivation and intervention adherence among patients. However, existing work often lacks a structured assessment of user experience over a longer period of time.

**Objective:**

This study aimed to assess the feasibility, user experience, and preliminary effectiveness of a VR-based rehabilitation program designed for patients with stroke to train upper limb and cognitive functions.

**Methods:**

Thirty-two chronic (n=19, 59%) or postacute (n=13, 41%) patients with stroke (mean age 60, SD 11 years) were enrolled. All participants performed 4 weeks of training, performing exercises in the Virtual Supermarket for Stroke (VSS). The VSS is an ecological VR-based application allowing customization of difficulty to make the task of “doing the shopping” more challenging throughout the sessions. Subjective outcomes were assessed after the first and last sessions. Clinical scales were administered at baseline and at the end of the treatment.

**Results:**

Of 32 participants, 31 (97%) completed the training. Flow (median 4.56, IQR 3.94-4.72; 5-point scale), sense of presence (Spatial Presence: median 3.44, IQR 12.85-3.85; Engagement: median 3.79, IQR 3.26-4.191; Naturalness: median 3.60, IQR 3.20-4.40; all 5-point scales), and affective state-related variables (Positive and Negative Affect Schedule; Positive Affect: median 4.60, IQR 4.00-5.00; Negative Affect: median 1, IQR 1.00-1.00) were satisfactory after the first session. Perceived ease of use was rated as very high (median 6.75, IQR 6.00-7.00; 7-point scale). No severe symptoms of cybersickness were recorded (Simulator Sickness Questionnaire [SSQ-TS]: median 11.22, IQR 0-20.57). At the end of the intervention, no significant differences were recorded in any subjective variable. Regarding clinical outcomes, significant improvements were recorded in balance (Berg Balance Scale pre: median 30, IQR 14.00-45.75; post: median 33.5, IQR 17.00-47.00; *P*=.02), upper limb motor functions (Motricity Index pre: median 45, IQR 15.25-69.00; post: median 46, IQR 32.00-77.00; *P*=.02; Box and Block pre: median 0, IQR 0-11.5; post: median 0, IQR 0-28; *P*=.005), and functional mobility (Time Up and Go pre: median 18, IQR 13.25-34.50; post: median 14, IQR 9.00-26.00; *P*=.005). No significant differences were recorded for general cognitive abilities (Mini-Mental State Examination pre: median 27, IQR 25-28; post: median 28, IQR 26-29), spasticity, and pain (visual analog scale pre: median 0, IQR 0-3.5; post: median 0, IQR 0-2).

**Conclusions:**

The study showed the preliminary feasibility of a rehabilitation program using the VSS. It addressed the essential topic of assessing VR-based rehabilitation user experience throughout the entire training period, shedding light on the features that can contribute to an optimal psychological experience. Clinical outcomes suggested that the VSS promoted neuroplasticity and that the recorded improvements could translate into meaningful functional gains in daily activities. Further studies with larger samples and patients with more severe disabilities are needed to confirm these results.

## Introduction

Stroke is the second leading cause of death and the third leading cause of disability worldwide [1,2], and the number of people affected by stroke has doubled in the last 30 years [2]. The majority of patients with stroke survive after the acute event and live with deficits in the motor, sensory, cognitive, mood, and behavioral domains for many years [3,4]. The severity of such deficits and the rate of recovery may vary depending on the type, location, and extent of the brain lesion [5]. The burden of disability after stroke is very high due to economic consequences on health care, social care, and loss of productivity.

Therefore, research and therapies aimed at improving the quality of life of patients with stroke are crucial [6]. Even though most recovery seems to occur during the first few months after stroke, patients can obtain clinical improvements even several months after the event [7]. Current literature suggests that positive outcomes can be achieved through functional training and pharmacological treatments; however, contrasting results [8] and existing barriers to implementing evidence-based recommendations [9] underscore the need for a deeper understanding of stroke rehabilitative treatments and their effects [6,8]. In addition, an open point remains on the administration of the intervention: it is currently unclear what the optimal dosage, frequency, and duration of the proposed therapy are, especially considering the variability and severity of symptoms that patients with stroke may exhibit, as well as the time from the event [9].

Although precise guidelines have yet to be defined, it is currently agreed that adherence to the proposed intervention is essential. The importance of continuing to exercise has been emphasized in several studies, which have highlighted the potential of intensive, task-specific, and meaningful exercises to recover sensorimotor functions [10,11]. Nonetheless, the average adherence rate to therapists’ recommendations after discharge ranges between 40% and 70% [12].

One of the solutions that researchers have explored to improve patients’ adherence and effectiveness of nonpharmacological interventions is the employment of new technologies, among which virtual reality (VR) has become one of the most popular [10,11,13]. VR is a medium that immerses the user in computer-generated scenarios and enables interaction with the surroundings. It has several characteristics that make it suitable for implementing or supporting rehabilitation interventions [11]: it allows the simulation of ecologically valid tasks, facilitates the transfer of acquired abilities to real life, controlled repetitions, and easy customization of difficulty [14]. Furthermore, VR has been shown to promote neuroplasticity and brain reorganization, thus enhancing recovery [15]. Additionally, it can provide real-time feedback that helps patients regain awareness of their performance [14] and promote training in potentially unsafe situations (eg, road crossing) that would not be feasible in reality [16].

Recently, therapeutic VR-based applications have begun to be assessed in the context of the concept of “flow.” Flow is a term used to describe a subjective psychological state that people experience when they are fully engaged in an activity to the point of losing track of time and their surroundings, except for the activity itself [17]. The concept of flow was first conceptualized by Csikszentmihalyi et al [17], which described it as an “optimal experience” characterized by 9 key features: *challenge-skill balance* (the activity difficulty matches the individual’s ability); *action-awareness merging* (actions feel automatic and effortless); clear goals; *unambiguous feedback* (feedback guides performance); *concentration* on the task; *sense of control* (the user can influence the situation or outcomes); *loss of self-consciousness; transformation of time* (time may seem to speed up or slow down); and *autotelic experience* (the activity is intrinsically rewarding and enjoyable).

The experience of flow was first assessed in sport, educational, and work contexts, as it was noted that positive psychological flow states are correlated with better performance. Nonetheless, it has been acknowledged that the flow concept applies well to applications developed with therapeutic purposes [18]. VR-based applications for rehabilitation must have a clear goal, provide immediate feedback, and the level of difficulty can often be customized. Additionally, these applications often incorporate gamified elements to maintain high levels of attention and challenge.

The systematic assessment of flow in the rehabilitative context could represent an opportunity. First, it would enable the determination of whether a specific VR application can promote an optical psychological experience, thereby indicating that it is usable, acceptable, and meaningful, ultimately leading to increased treatment compliance [19,20]. Second, it could suggest that it has the potential to enhance the therapeutic efficiency [18].

Although the evaluation of flow and user experience in general is often performed in sports [21,22] and work-related contexts [23], it is generally neglected or lacks analytical rigor in clinical studies [24,25]. A few studies observed a state of flow in their participants but did not assess it with appropriate tools [26]. A review conducted in 2021 analyzed the assessment of flow in VR applications developed for neurorehabilitation; the authors were able to trace only 10 studies [18]. More recently, Saric et al [27] and Pastore-Wapp et al [28] used flow to evaluate the user experience of individuals with Parkinson disease who underwent VR-based training for hand dexterity. Geiser and colleagues [29] assessed flow in patients with left neglect due to stroke who were trained with auditory motion stimulation for 3 weeks. In all cases, the positive experience was considered to be associated with the enjoyment of the experience and the motivation to continue using VR.

This work is positioned in this context by aiming to assess the feasibility, flow, and user experience of patients with stroke during a 4-week intervention with an immersive VR application specifically developed to support cognitive functions and upper limb rehabilitation. The decision to develop an ad-hoc application was made to avoid the limitations commonly associated with commercial games, including inaccessible interactions for people with disabilities, a lack of ecological validity, and difficulty in customization [30]. The immersive VR application object of this work is derived from previous work carried out by our research group in previous years. In particular, we started with the development and validation of a virtual supermarket dedicated to the training of visuo-spatial abilities in which people could walk naturally; we assessed and demonstrated its usability and acceptance in a group of healthy young adults [31] and in older adults with mild cognitive decline or subjective cognitive decline [32]. Later, we started developing a second version with a simplified interaction, which was tested in the healthy population with positive results [33]. The application we used in this study, namely the Virtual Supermarket for Stroke (VSS), represents the continuation of this research line and, besides the simplified interaction, was specifically designed to reproduce an activity of daily living and to be customized to increase the level of difficulty throughout the 4 weeks of training.

We hypothesized that the chance of experiencing an activity of daily living (ADL) with a customized and adaptable level of difficulty could help maintain the flow levels of the exercise, as well as have other positive effects, and positively impact the sense of presence [22] and emotional state. Moreover, we expected the intervention to be feasible, with patients being able to interact with the application and not experiencing any adverse symptoms due to cybersickness.

Although this study was designed as a user-experience study, a secondary aim was to assess the effectiveness of the proposed VR-based intervention at the clinical level via standard clinical scales.

## Methods

### Ethical Considerations

The study obtained ethical clearance from the Insubria Ethical Committee (reference No. 70, December 1, 2020) and complies with the Declaration of Helsinki. All participants signed written informed consent before being enrolled; this consent also included permission to take pictures and record videos. To ensure the participants’ privacy, data were collected in a pseudonymized form, and the keys were available only to the clinical personnel involved in the study. Also, any images included in the manuscript have been carefully reviewed to ensure that no individual can be identified. Participants did not receive any compensation for taking part in the study. This study adhered to the RATE-XR (Rationale, Accessibility, Training, and Effectiveness for Extended Reality) checklist [34] (Checklist 1).

### Participants

Participants were recruited through convenience sampling among patients with stroke referred to the Villa Beretta Rehabilitation Center (Costa Masnaga, LC, Italy) at the Rehabilitation Department of Valduce Hospital, where the study was carried out. Participants were either in the postacute (time from the stroke >15 d) or chronic phase (>6 mo). All met the following inclusion criteria: age 18 years or older; clinical stability; absence of pain, postural instability, muscle hyperactivity, and impairments that prevent the accomplishment of the reaching task; Mini-Mental State Examination (MMSE) 20 or an equivalent cognitive level for patients with aphasia.

Clinical stability, particularly for individuals with subacute poststroke, was defined as a period of 2 to 4 weeks poststroke during which patients completed acute hospitalization and were medically stable. This choice allowed for an optimal enrollment window of 1 to 3 months post stroke, where there is a balance between medical stability and the neuroplasticity window.

The cutoff set for MMSE score was selected based on several considerations. First, MMSE scores 20 or higher (≥20) indicate sufficient cognitive capacity to understand VR task instructions, safety protocols, and provide informed consent for participation in experimental technology [35,36]. Second, MMSE scores below 20 (<20) have been associated with reduced rehabilitation potential and motor learning capacity in stroke populations, which could limit the ability to benefit from VR-based interventions [37,38].

Third, VR rehabilitation requires intact working memory, sustained attention, and executive function to engage with immersive environments and process multimodal feedback—cognitive domains that are relatively preserved in individuals with an MMSE score of 20 or higher [39,40]. Finally, this cutoff is consistent with previous VR stroke rehabilitation trials [41,42] and minimizes floor effects on secondary cognitive outcome measures.

Exclusion criteria were a history of seizure or motion sickness, severe visual deficits, and inability to provide informed consent. The sample size was calculated using the formula reported by Candel and van Breukelen [43] for single-arm pre-post studies, and considering flow (specifically Short Flow Scale; see Outcomes section) as the main outcome. Given previous experiences and available literature on the topic [44], the required sample was 23 participants, using SD=1.2, α=.05, power=80%, and expecting a pre-post variation equal to 0.65. The final sample size was increased to 30, considering the possibility of 20% dropouts and aligning the final sample to other studies assessing feasibility [45].

### Equipment

**Figure 1.**
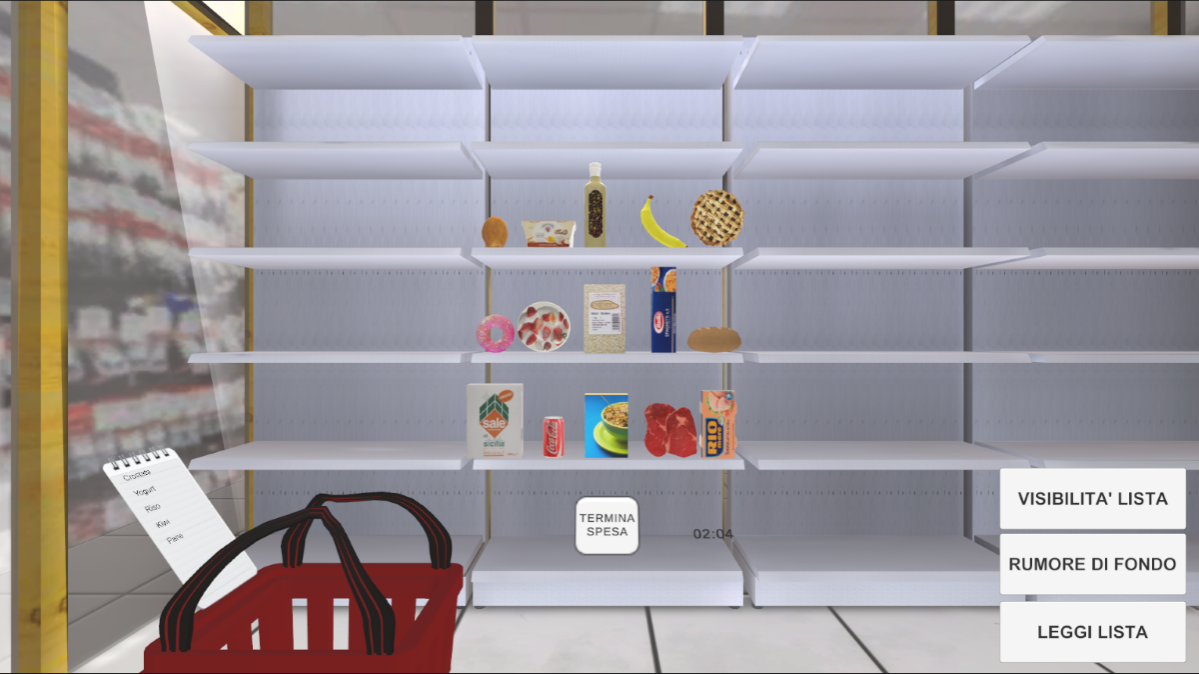
A screenshot of the aisle environment of the Virtual Supermarket for Stroke. To complete the shopping, the button “end shopping’' [termina spesa] has to be clicked. Buttons at the bottom-right corner are available to the therapist to change the visibility of the list [visibilità lista], to turn the background noise on/off [rumore di fondo], and to replay the list items [leggi lista]. The basket could be placed on the right or the left side, depending on the upper limb to train [46].

**Figure 2.**
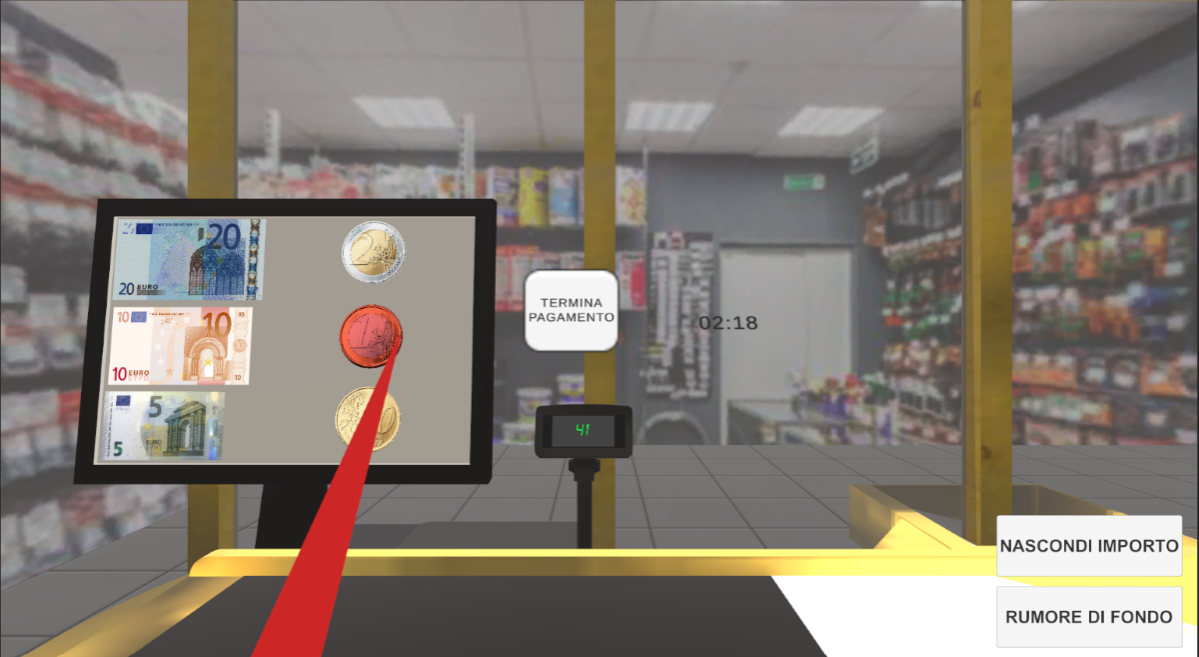
A screenshot of the payment environment of the Virtual Supermarket for Stroke. The [termina pagamento] serves to end the selection of banknotes and coins. Buttons at the bottom right corner are available to the therapist to change the visibility of the amount to pay [visibilità importo] and to turn the background noise on/off [rumore di fondo] [46].

The VSS is an immersive VR application dedicated to the rehabilitation of stroke patients. It was developed with Unity and deployed for Oculus Rift v2. The VR environment is constituted by 2 scenarios. The first is devoted to picking groceries from the shelf (Figure 1); the second is paying for such items (Figure 2). Both these tasks can be performed while staying seated to ensure all participants’ safety and make the VSS accessible to patients in wheelchairs, too.

A series of features is available to therapists to customize the difficulty of training throughout the rehabilitative path. Before starting the exercises, the therapist can adjust the position (right/left) of the shopping basket, the number of items on the shelves (9 or 15), the number of target items (ie, items to be picked from the shelves) on the shopping list (from 1 to 8), and select if grocery items are shown on the list with their names or as categories (eg, dairy products, dishware, vegetables); in this second case, the task is to pick all the items corresponding to such categories. Moreover, during the exercise, some buttons (visible only on the therapist’s PC screen) allowed the therapist to play a background noise, which served as a distracting sound, and to hide or show the shopping list or the amount of money to be paid. When the shopping list was hidden, the therapist could play a clip (created via text-to-speech) indicating to the patient the grocery items to pick. All the therapists/psychologists involved in the study were trained by the VSS developer to set up the game area and use the application, which was developed by the researchers who created the VSS.

All the interactions within the VR environment occurred using the Oculus controller. The grocery items could be picked using a direct interaction, that is, reaching for the item and pressing the controller grip button to hold it and transport it around. All the other interactions (ie, coin selection and button press) occurred using a ray interactor (Figure 2) [47].

At the end of each task (item grabbing and payment), a panel was shown to the patients reporting their performance (ie, the time needed to complete the task, the errors, the omissions, or the amount of money still to pay).

Given its features, the VSS was expected to promote the retraining of motor and cognitive functionalities, in particular, the upper limb task-related reaching movement, a goal-directed, functional movement carried out in a natural environment [48], and visuospatial search, memory, and action planning.

### Protocol

Each patient underwent 12 sessions of 20 minutes, in which the tasks of doing the shopping and paying at the cash register were repeated recursively. The therapist chose the level of difficulty, the presence of distractors, and other elements that increase the complexity of the task, both at the beginning and during the session. All sessions occurred in a quiet room, where only the patient and the therapist were present. Upon arrival, patients using a wheelchair were guided into the game area, and their wheelchair was braked. Patients who walked or used crutches were accommodated in a chair. All were then helped to wear and adjust the head-mounted display. Patients completed the task with the impaired arm whenever possible; if their motor functions were too low on the impaired side, they used the other upper limb.

During the first session, each participant was presented with the VSS and the tasks to perform, along with an explanation of the interaction modalities. All performed the first session at the lowest level of difficulty. After 20 minutes of exercise, the psychologist supervising the session administered paper-based questionnaires evaluating their user experience. The same questionnaires were administered again to all patients immediately after the last session. In the case of patients with aphasia, questionnaires were answered by pointing.

Patients were also administered the clinical scales presented in the Outcomes section at baseline and after completing the last exercise session.

### Outcomes

The user experience was evaluated by administering a series of questionnaires at the end of the first and last sessions. Questionnaires were administered by a psychologist, and answers were collected on paper. The collected questionnaires were:

1. The Short Flow Scale (SFS) [49], which uses 9 items to measure *flow* (ie, a measure of absorption in what one is doing and how much the subjective experience is optimized);
2. The International Test Commission—Sense of Presence Inventory (ITC-SOPI) [50], which evaluates the Sense of Presence (ie, the sense of “being there” in a computer-generated environment along 4 subscales: spatial presence, engagement, naturalness, and side effects);
3. The Positive and Negative Affect Schedule—Short Form [51] for the assessment of positive and negative traits and states (ie, joy, high levels of energy, concentration, and distress, anger, contempt, or fear, respectively);
4. The Simulator Sickness Questionnaire (SSQ) [52] to assess cybersickness according to Nausea (SSQ-N), Oculomotor disturbance (SSQ-O), and Disorientation (SSQ-D), and general score (SSQ-TS); and
5. The Technology Acceptance Model (TAM3) subscale for the evaluation of perceived ease-of-use (PEOU) [53].

SFS, ITC-SOPI, and PANAS were assessed on a Likert scale ranging from 1 (strongly disagree) to 5 (strongly agree), whereas PEOU was evaluated with a scale ranging from 1 to 7 (maximum perceived ease of use); within SSQ, the occurrence of each symptom was assessed from *not at all* to *severe* on a 4-point scale.

To evaluate whether the proposed intervention brought clinical improvements, the following clinical scales were administered before and after the training period:

1. MMSE [54] or an equivalent for patients with aphasia: MMSE is a brief 30-point questionnaire used to screen general cognitive impairments. It includes orientation, immediate memory, attention, calculation, recall, and language tasks; it represents a common instrument to assess cognitive changes.
2. Visual analog scale for pain [55]: the scale consists of a 10 cm horizontal line going from 0 (no pain) to 10 (worst imaginable pain). It represents a quick instrument to subjectively assess pain, which, in stroke, is mostly investigated from a clinical diagnosis perspective rather than from the patient’s perspective [56].
3. Motricity Index (MI) [57]: it is a clinical scale used to assess limb motor function, especially in individuals with stroke. It measures muscle strength in the upper limb (pinch grip, elbow flexion, and shoulder abduction) and lower limb (ankle dorsiflexion, knee extension, and hip flexion). Each movement is scored on a 6-point scale (0‐33), based on the degree of movement and resistance against gravity.
4. Modified Ashworth Scale [58] for spasticity on arm abductors and pronator, elbow flexor and extensor, wrist flexor and extensor, and finger flexors (flexor digitorum superficialis and profundus, and flexor pollicis longus): an examiner performs it by moving a limb passively through its range of motion and grades the resistance felt in the muscle from 0 (no increase in muscle tone) to 4 (rigid flexion or extension).
5. Box and Block test (BnB) [59] with both arms (affected [A] and nonaffected [NA]): it constitutes a measure of manual dexterity, and it is computed as the number of wooden blocks (2.5 cm) transferred from one compartment to another in 1 minute.
6. Berg Balance Scale [60]: it consists of 14 functional balance tasks that are rated by the examiner from 0 (unable to perform) to 4 (performs independently and safely). The maximum score is 56; scores below 45 indicate risk of falls.
7. Timed Up and Go test [61]: it is used to assess mobility, balance, and fall risk. The examinee starts seated, stands up, walks 3 m, turns around, and walks back to return seated. The time taken to perform the task constitutes its score.

### Statistical Analysis

To assess the randomness of missing data, we used the Little’s Missing Completely At Random (MCAR) test. Multiple imputation was performed as a sensitivity analysis to evaluate the robustness of the results with respect to missing data handling.

The reliability of the questionnaires’ structure was calculated with the McDonald Ω index; a frequently cited acceptable range of the Ω coefficient is a value of 0.70 or higher [62,63]. The normality of the data distribution was assessed using the Kolmogorov-Smirnov test. Descriptive statistics are used to describe the outcomes. In the case of nonnormal distribution, the 95% CIs estimated via bootstrapping (2000 resamples) pre-post differences were assessed via Wilcoxon signed rank test. In all analyses, statistical significance was set at *P* less than .05.

## Results

### Participants and Missing Data

**Figure 3.**
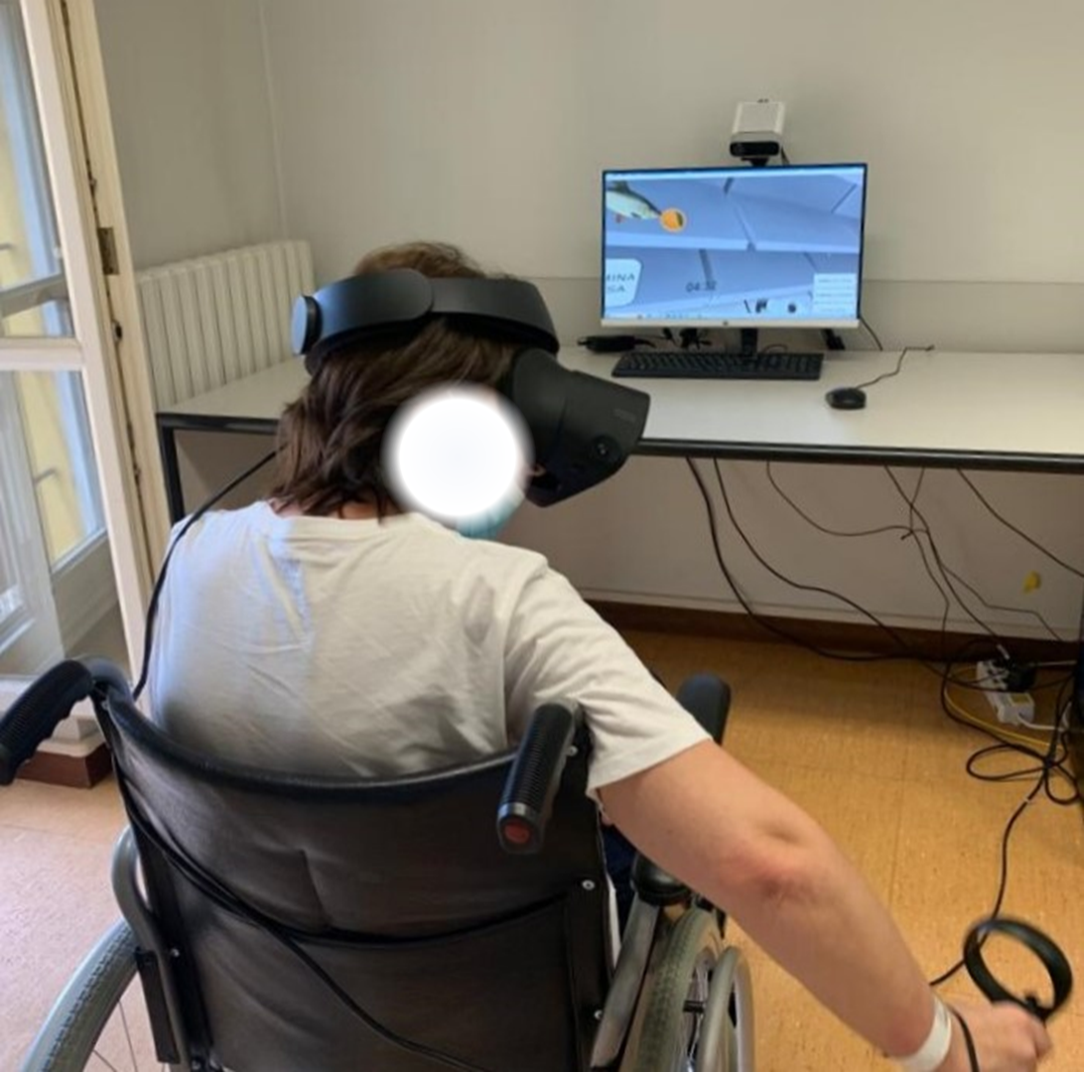
One of the participants during a Virtual Supermarket for Stroke session.

Thirty-two patients with a mean age of 60 (SD 11) years were enrolled in the study. One participant experiencing the VSS is shown in Figure 3. The median time from the stroke event was 7 (IQR 16, min: 16 d, max: 11 y) months, with 13 participants being in the subacute phase and 19 in the chronic stage. Twenty-three participants had right-brain damage. Demographic and baseline data of the study participants are presented in Multimedia Appendix 1.

All patients except one completed the training; no adverse events were recorded. One participant left the study because they did not feel familiar with and did not feel at ease while interacting with technology. For this participant, only clinical scales were collected at T1, leading to user experience–related results being calculated on n=31. There were no other missing data.

Missing data in the clinical scales were evaluated by recoding nonparticipation cases (eg, patients unable to walk could not perform the Time Up and Go test at any time point) as valid scores. Considering this, the overall proportion of missing data was approximately 4%, with up to 12% missingness in 5 clinical scales. The Little’s MCAR test was not significant (*χ*^2^_85_=104.308; *P*=.07), indicating that other data were missing completely at random. Using multiple imputation as a sensitivity analysis, results were highly consistent across approaches, indicating that missing data did not materially influence the findings. Therefore, complete-case data were analyzed.

### User Experience

**Figure 4.**
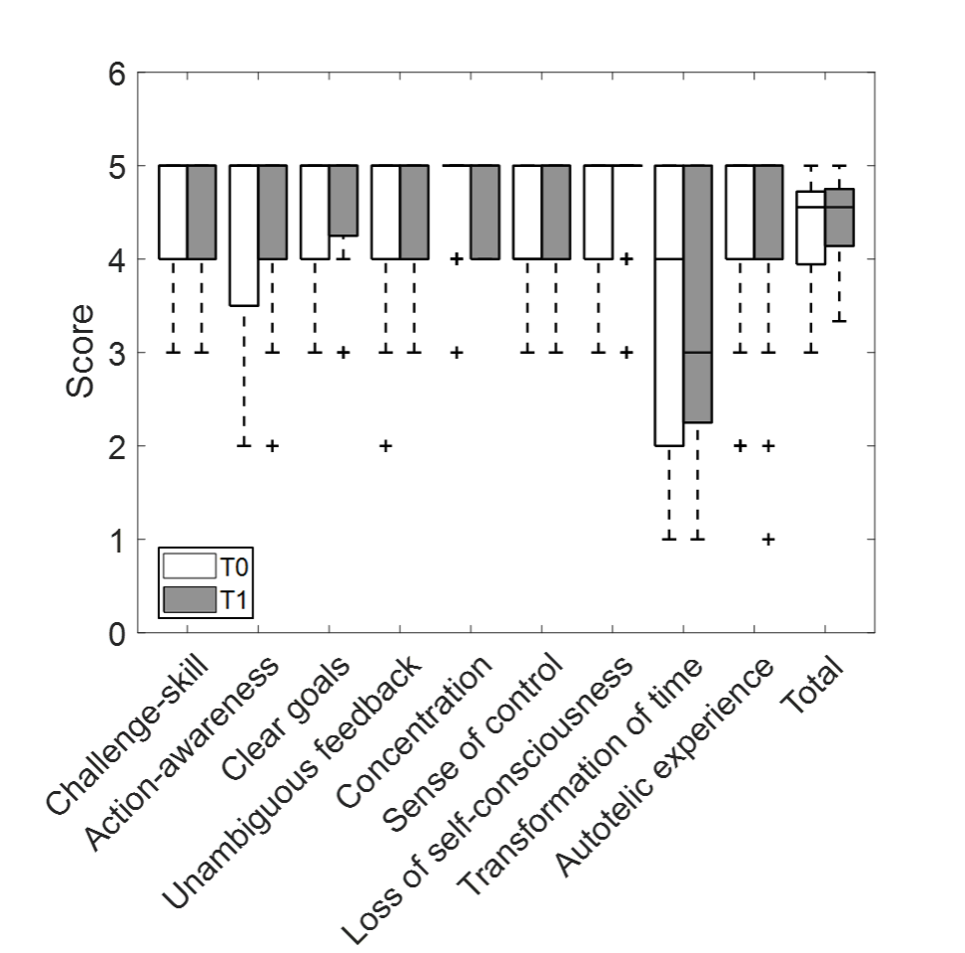
Flow subscales collected from participants with stroke using the Virtual Supermarket for Stroke in the pretest-posttest study. Data are shown as boxplots, where whiskers extend to the most extreme values within 1.5 times the IQR and individual points denote outliers. Measurements were performed at T0, after the first session, and at T1. T1 was collected after 4 weeks of training, 3 times per week, at the end of the 12th session.

The results obtained for all flow subscales are reported in Figure 4; there are no statistically significant differences for any of them (*P*>.05). The increase in the total flow score was almost significant (*P*=.05).

Descriptive statistics for the user experience questionnaires investigating presence, positive and negative affect, cybersickness, and perceived ease of use are reported in Table 1.

**Table 1.** Descriptive statistics of user experience-related variables collected from participants with stroke using the Virtual Supermarket for Stroke in the pretest-posttest study.^a^

	Pre							Post						
	Median (IQR)	Min	Max	95% CI	Ω	Deleted items	K-S test (*P* values)	Median (IQR)	Min	Max	95% CI	Ω	Deleted items	K-S test (*P* values)
Flow	4.56 (0.64)	3	5	4.33-4.67	0.79	—^b^	.01^c^	4.57 (1.00)	3	5	4.33-4.67	0.64	7, 8	.007^c^
SP^d^	3.44 (1)	1.59	4.82	3.15-3.71	0.87	—	.01^c^	3.71 (1.18)	1.41	4.88	3.16-3.80	0.89	—	.18
ENG^e^	3.79 (1)	2.17	4.92	3.45-3.90	0.74	5	.12	3.83 (1.00)	1.5	4.75	3.54-4.08	0.89	5	.048^c^
NAT^f^	3.60 (1.60)	2	5	3.59-4.08	0.75	27	.16	4.00 (1.80)	1.2	5	3.20-4.20	0.82	—	.02^c^
PA^g^	4.60 (1)	1.40	5	4-4.80	0.94	—	<.001^c^	4.60 (0.80)	3.8	5	4.20-5	0.63	—	<.001^c^
NA^h^	1 (0)	1	3	1-1	0.93	—	<.001^c^	1 (0)	1	2	1-1	0.84	—	<.001^c^
SSQ-N^i^	0 (4.77)	0	104.94	0-0	0.91	—	<.001^c^	0 (7.16)	0	28.62	0-0	0.71	1	—
SSQ-O^j^	15.16 (22.74)	0	75.8	7.58-22.74	0.78	—	<.001^c^	7.58 (15.16)	0	68.22	0-15.16	0.95	1	—
SSQ-D^k^	13.92 (27.84)	0	55.68	0-27.84	0.82	—	<.001^c^	0 (27.84)	0	55.68	0-13.92	0.92	5, 8	<.001^c^
SSQ-TS^l^	11.22 (20.57)	0	112.2	0-16.83	0.94	—	<.001^c^	7.48 (14.96)	0	48.62	0-14.96	0.72	Nausea	<.001^c^
PEOU^m^	6.75 (1)	3.75	7	6-7	0.94	—	<.001^c^	6.75 (1)	3.8	5	6.25-7	0.85	—	<.001^c^

a Data were collected at T0 (pre), after the first session, and at T1 (post). T1 was collected after 4 weeks of training, 3 times per week, at the end of the 12th session. Data is presented as median values with IQRs, 95% CIs, and minimum and maximum values. Results of the Ω test for reliability and the Kolmogorov-Smirnov test are also reported.

b Not applicable.

c Indicates statistical significance (*P*<.05).

d SP: spatial presence.

e ENG: engagement.

f NAT: naturalness.

g PA: positive affect.

h NA: negative affect.

i SSQ-N: Simulator Sickness Questionnaire—Nausea.

j SSQ-O: Simulator Sickness Questionnaire—Oculomotor Disturbance

k SSQ-D: Simulator Sickness Questionnaire—Disorientation.

l SSQ-TS: Simulator Sickness Questionnaire—General Score.

m PEOU: perceived ease of use.

No significant difference was found between pre- and postassessment regarding negative effects, engagement, realism, spatial presence, ease of use, and positive and negative affects. No significant difference was found for nausea, oculomotor, and disorientation symptoms or the total score of the Simulator Sickness Questionnaire.

### Clinical Outcomes

**Figure 5.**
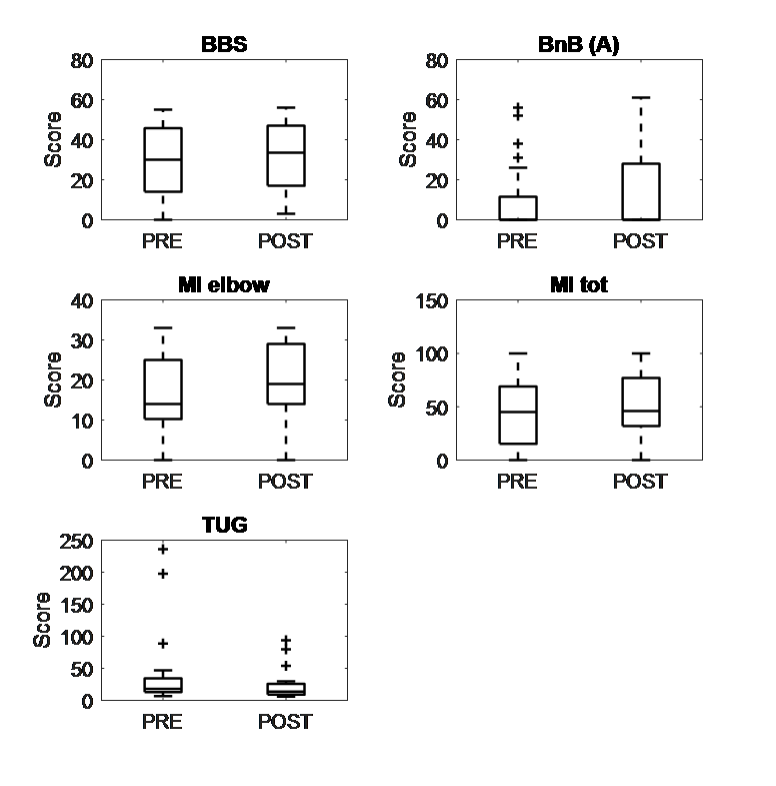
Boxplots of the clinical scales collected from participants with stroke using the Virtual Supermarket for Stroke (VSS) in the pretest-posttest study. Whiskers extend to the most extreme values within 1.5 times the interquartile range, and individual points denote outliers. The reported scales are the ones that showed a significant (*P*<.05) improvement postintervention (ie, after 4 wk of training, the VSS was used three times a wk). BBS: Berg Balance Scale; BnB (A): box and block (affected side); MI: Motricity Index; TUG: Time Up and Go.

The measures of clinical outcomes before and after the 4 weeks of intervention are reported in Table 2. Significant improvements were recorded for the affected arm in the Box and Block test, MI (elbow and total scores). Moreover, patients improved their balance and their performance in the Time Up and Go test. Significant differences are shown in Figure 5.

**Table 2.** Descriptive statistics of the clinical scales collected from participants with stroke using the Virtual Supermarket for Stroke in the pretest-posttest study.^a^

	Pre		Post		*P* value
	Median (IQR)	95% CI	Median (IQR)	95% CI	
MMSE^b^	27 (3)	25-28	28 (3)	27-29	.15
VAS^c^	0 (3.50)	0-2	0 (2)	0-1	.35
BBS^d^	30 (31.80)	20-37	33.50 (30)	23-44	.02^e^
MAS^f^					
Arm abductor	0 (0)	0-0	0 (0)	0-0	.50
Arm internal rotation	0 (0.50)	0-0	0 (0.75)	0-0	.94
Arm pronator	0 (0.50)	0-0	0 (0.75)	0-0	>.99
Elbow extensor	0 (0)	0-0	0 (0)	0-0	.94
Elbow flexor	0 (1)	0-1	0 (1.38)	0-1	.50
Wrist extensor	0 (0)	0-0	0 (0)	0-0	>.99
Wrist flexor	0 (0.50)	0-0	0 (1)	0-0	>.99
Finger extensor	0 (0)	0-0	0 (0)	0-0	>.99
FDS^g^	0.50 (1.75)	0-1.25	1 (1.38)	0-1.25	.38
FDP^h^	0 (1)	0-0.50	0 (1)	0-0.50	>.99
FPL^i^	0 (1)	0-0	0 (1)	0-0	.28
BnB (A)^j^	0 (11.50)	0-29	0 (28)	0-41	.005^e^
BnB (NA)^j^	44 (15.50)	31-45	50.50 (13.50)	29-51	.09
MI^k^ (pinch)	11 (25)	0-19	19 (20.50)	11-26	.10
MI (elbow)	14 (14.80)	14-19	19 (15)	14-25	.02^e^
MI (shoulder)	14 (14.50)	9-14	14 (16)	14-25	.19
MI tot	45 (53.75)	24-59	46 (45)	37.50-75	.02^e^
TUG^l^ (s)	18 (21.25)	14-34	14 (17)	10-23	.005^e^

a Data were collected at T0 (PRE), after the first session, and at T1 (POST). T1 was collected after 4 weeks of training, 3 times per week, at the end of the 12th session. Data is presented as median values with interquartile ranges and 95% confidence intervals estimated via bootstrapping (2000 resamples).

b MMSE: Mini-Mental State Examination.

c VAS: visual analog scale.

d BBS: Berg Balance Scale.

e Indicates statistical significance (*P*<.05).

f MAS: Modified Ashworth Scale.

g FDS: flexor digitorum superficialis.

h FDP: flexor digitorum profundus.

i FPL: flexor pollicis longus.

j BnB: box and block test with affected (A) and nonaffected (NA) arm.

k MI: Motricity Index.

l TUG: Timed Up and Go.

## Discussion

### Main Findings

This study presents a feasibility assessment of a VR-based intervention for the rehabilitation of cognitive abilities and upper limb functions in patients with stroke, focusing on user experience and preliminary effectiveness. In particular, we assessed that the ecological environment and the features of the VSS application promoted an optimal psychological experience, enhancing flow and thereby increasing motivation and adherence throughout the entire duration of the training. We found that the general experience was positive, and participants welcomed the possibility of rehabilitation with the support of immersive technologies, which also led to some improvements in functional outcomes. Also, we demonstrated that the designed intervention was feasible, with no side effects and excellent adherence among stroke survivors (97%).

### Subjective Outcomes

The subjective outcomes collected after the first session showed satisfying levels, with flow and positive affect scores very close to the highest possible value. The same was recorded for PEOU, possibly indicating that the intuitive interaction with virtual objects in the VSS also mediated the positive experience. This finding aligns with a previous study that investigated the acceptance of immersive VR technologies among patients with stroke [30,64].

The choice of a simple interaction methodology was effective and made possible by the use of an ad-hoc, developed application. In fact, the application’s features made the solution easily accessible, even for patients with motor limitations. On the contrary, the use of a commercially available application, which would have possibly provided more varied and engaging scenarios, would have required checking it for customization settings to enable effective interaction for patients with stroke [65].

Presence-related scales were all satisfactory; thus, patients felt present and recognized the environment as an ecological and familiar one. Conversely, negative affect was low, and median cybersickness scores were all within the acceptable range (≤20) [52]. This confirmed the outcomes obtained before with slightly different versions of the Virtual Supermarket and different populations (healthy volunteers [33] and older adults with cognitive decline [32,66]). In particular, in this case, we recorded almost no nausea-related symptoms; instead, the symptoms were mostly present in the oculomotor and disorientation domains, as more typically occurs in nonnavigational environments [67].

At the end of the period of trial, all the subjective outcomes preserved the same satisfactory and, in some cases, more than satisfactory trends. This demonstrated that the design choice we made for the application effectively addressed the maintenance of flow throughout the experience, that is, we could balance patients’ skills and the challenge of the task, and provide an engaging scenario in which they could feel focused and immersed. Also, the obtained outcomes confirmed our hypothesis that introducing some challenging elements (ie, the possibility of customizing the levels of difficulty), addressing different cognitive abilities (eg, attention, memory), and the visualization of the performance could contribute to maintaining the experience engaging and able to induce positive feelings, even over a longer period of time.

These features represent essential points in the field of rehabilitation, in which prolonged, or even lifelong, interventions are often essential to counteract limb disuse or misuse, or cognitive decline [68]. As already mentioned, these variables and flow, in particular, have been explored in sport and other disciplines because of their positive association with performance, positive experience, motivation, and enjoyment [18]. Despite this, its assessment remains sparse in the health sector, which may negatively impact treatment compliance and the subsequent effectiveness of proposed VR-based interventions [65].

This has also been shown in a review performed in 2020, which focused on user experience in general and immersive VR; it showed that research in the field of human-immersive VR interaction still presents some methodological and technological gaps [69]. When examining the rehabilitation field, such gaps are even more evident: of the 65 articles included in the review, only four involved patients, and 35 included participants only in their 30 s.

Future studies should thus consider tackling the user experience assessment more broadly to evaluate patients’ needs not only from a clinical perspective but also from a subjective and intrinsic motivation point of view [65,70].

### Clinical Outcomes

Regarding clinical outcomes, we observed positive results in various clinical scales. We recorded a significant improvement in the Berg Balance Scale and the Timed-Up-and-Go test, indicating increased trunk control in all patients. Moreover, considering the entire sample, we recorded an increase in the impaired limb scores, with significant changes in the Box-and-Block and otricity Index (total and elbow score) tests.

The observed improvements in upper limb dexterity (Box-and-Block Test) and motor strength (MI) suggest that the intervention may translate into meaningful functional gains in daily activities such as reaching, grasping, and manipulating objects [71]. The enhanced balance and mobility indicate potential benefits for overall independence and fall prevention [72].

These changes were recorded even if some patients performed the task with the nonimpaired arm, possibly suggesting that the improvements were mostly linked to better trunk control and action planning (which have recently been reported to be related [73]) rather than the arm motor function improvement per se. To this aim, the fact that the supermarket simulated an activity of daily living may have also contributed positively to the rehabilitation of executive functions [74]. Indeed, the ecological validity of shopping tasks has been previously verified in studies that utilize this scenario to assess and train executive functions and action planning, in particular [75].

The intervention proposed using the Virtual Supermarket frames within the task-oriented training. In fact, it emphasizes functional activities and patient involvement. Furthermore, it includes task repetition, active participation, and the modulation of the training intensity. From a clinical perspective, these interventions—even those supported by VR—have demonstrated effectiveness in enhancing limb function and balance, as well as promoting neuroplasticity [76,77].

Therefore, it is possible that, in our case as well, the modulating effects of neuroplasticity allowed us to observe clinical improvements even in chronic patients exercising with the less-affected limb [78,79]. Unfortunately, the small sample size, defined for the assessment of flow, did not allow us to explore subgroup differences further (ie, considering time from the stroke event, in the postacute or chronic phases, or the use of the affected or nonaffected side). However, the positive results should encourage the conduct of more structured, clinical-oriented trials to unveil potential indications for future intervention characterization. In fact, the recorded improvements across multiple functional domains support the potential clinical relevance of the intervention. The effects of the proposed intervention on both upper limb function and balance suggest that it could be integrated into standard rehabilitation protocols to promote more holistic recovery.

### Limitations

All the discussed results must be treated cautiously. We are aware that the current study presented some limitations that prevented the generalization of the results. First, the sample size was small and estimated based on the assessment of our main outcome (ie, flow); thus, the statistical power may not be sufficient to draw conclusions at the clinical level. Second, the population was heterogeneous, including both patients in the sub-acute and chronic phases who completed the exercise with either the impaired or less-impaired side. Finally, we did not have a control group. Nonetheless, the study allowed for assessing the user experience in a systematic and multidimensional way and highlighted the potential of a customizable, ecological, and immersive VR-based application for the rehabilitation of motor functions in patients with stroke.

### Conclusions

This work addressed an important aspect in the context of VR-assisted rehabilitation, as user experience is generally evaluated on a single session or by focusing on a single aspect (eg, usability).

It contributed to the current body of knowledge on the use of immersive VR for stroke rehabilitation, providing insights into the features that helped maintain high flow levels and highlighting the importance of exploring the subjective domain in clinical trials as well.

From a clinical perspective, the intervention appears feasible and well-tolerated. Its simple setup and ease of use make it potentially adaptable to a variety of clinical settings, and potentially for future use at home or in unsupervised contexts (eg, with a stand-alone head-mounted display and automatic progression of difficulty).

In the future, it would be valuable to increase the difficulty levels and include different interaction technologies (eg, hand tracking) to make the application more accessible to patients with limited arm motor function, thereby providing a longer training period.

Moreover, as already mentioned, it would be essential to enlarge the sample of participants, thus allowing for a more in-depth investigation of the optimal phase for administering the exercise and, in general, of the clinical effectiveness of the VSS application.

## Supplementary material

10.2196/79584 Multimedia Appendix 1 Demographic and baseline data of the patients enrolled in the study. BBS: Berg Balance Scale; flex: flexion; BnB: box and block test with the dominant (D) and the nondominant arm (ND); ext: extension; FDP: flexor digitorum profundus; FDS: flexor digitorum superficialis; FPS: flexor pollicis longus; IR: internal rotation; MAS: Modified Ashworth Scale; MI: Motricity Index; MMSE: Mini-Mental State Examination; VAS: visual analog scale; TUG: Time Up and Go.

10.2196/79584 Checklist 1 RATE-XR checklist.

## Abbreviations

ITC-SOPI: International Test Commission—Sense of Presence Inventory
MI: Motricity Index
MMSE: Mini-Mental State Examination
PANAS: Positive and Negative Affect Schedule
PEOU: Perceived Ease of Use
RATE-XR: Rationale, Accessibility, Training, and Effectiveness for Extended Reality
SFS: Short Flow Scale
SP: spatial presence
SSQ: Simulator Sickness Questionnaire
TAM3: Technology Acceptance Model
TS: total score
TUG: Timed Up and Go
VR: virtual reality
VSS: Virtual Supermarket for Stroke
