# Supplementary material for: Feasibility and User Experience of Immersive Virtual Reality–Based Rehabilitation in Patients With Stroke: Single-Arm Pretest-Posttest Pilot Study
Source: JMIR Serious Games. 2026 Mar 11;14:e79584. doi: 10.2196/79584 (PMC12978540; doi:10.2196/79584)
Supplement: Multimedia Appendix 1 [file games-v14-e79584-s001.docx]

# Demographic and baseline data of the patients enrolled in the study

MMSE: Mini Mental State Examination; VAS: visual analog scale; BBS: Berg Balance Scale; MAS: modified Ashworth scale; BnB: box and block test with the dominant (D) and the non-dominant arm (ND); MI: Motricity Index; TUG: time up and go; flex: flexion; ext: extension; IR: internal rotation; FDS: flexor digitorum superficialis; FDP: flexor digitorum profundus; FPS: flexor pollicis longus.

| **ID** | **Age** | **Side** | **MMSE** | **VAS** | | **BBS** | **MAS** | **BnB (D)** | **BnB (ND)** | **MI** | | | | | **TUG** |
| --- | --- | --- | --- | --- | --- | --- | --- | --- | --- | --- | --- | --- | --- | --- | --- |
|  |  |  |  |  | |  |  |  |  | **pinch** | **elbow** | **shoulder** | **tot** | **(s)** | |
| 1 | 55 | Left | 25 | 8 | 23 | |  | na | na | 0 | 0 | 0 | 1 | na | |
| 2 | 56 | Right | 30 | 0 | 55 | | elbow ext.: 1+ | 59 | 56 | 33 | 33 | 33 | 100 | 9 | |
| 3 | 39 | Left | 30 | 0 | 37 | | arm pronator: 1; finger ext.: 2; FPL: 1 | 53 | 0 | 11 | 19 | 14 | 45 | 32 | |
| 4 | 46 | Left | 29 | 0 | 54 | | FDS: 2 | 54 | 29 | 19 | 19 | 19 | 58 | 7 | |
| 5 | 69 | Left | 25 | 5 | 32 | | arm abductor: 1; arm IR: 1; arm pronator: 1+ | 46 | 0 | 0 | 14 | 19 | 34 | 27 (with cane) | |
| 6 | 37 | Left | 30 | 0 | 51 | | arm IR:1; elbow flex: 1; FDS: 1; FDP: 1 | 66 | 3 | 11 | 19 | 14 | 45 | 14 | |
| 7 | 56 | Left | 28 | 0 | 33 | |  | 40 | 31 | 26 | 25 | 25 | 77 | 9 | |
| 8 | 68 | Left | 28 | 0 | 3 | | elbow ext.: 1; wrist ext: 1; FDS: 1; FDP: 1 | 37 | 0 | 19 | 14 | 14 | 48 | na | |
| 9 | 58 | Right | 23 | 0 | 48 | |  | 54 | 52 | 33 | 33 | 33 | 100 | 23 (with cane) | |
| 10 | 58 | Right | 28 | 0 | 13 | |  | 43 | 0 | 0 | 0 | 9 | 10 | 236 | |
| 11 | 78 | Right | 29 | 4 | 42 | | — | 7 | 42 | 26 | 19 | 14 | 59 | 11 (with cane) | |
| 12 | 58 | Right | 28 | 1 | 28 | | arm abductor: 1+; arm IR: 2; elbow ext.: 3; elbow flex: 3; wrist flex:3; FDS:3 | 0 | 57 | 19 | 14 | 14 | 48 | 18 | |
| 13 | 66 | Left | 25 | 0 | 0 | | arm IR: 2; arm pronator: 2; elbow flex: 2; wrist flex: 3; FDS: 3; FDP: 2: FLP: 2 | 20 | 0 | 0 | 0 | 0 | 1 | na | |
| 14 | 66 | Left | 27 | 2 | 9 | | wrist flex: 2; FDS: 2; FLP:2 | 41 | 0 | 0 | 0 | 0 | 1 | 198 | |
| 15 | 55 | Left | 29 | 0 | 51 | | arm pronator: 1; elbow flex: 1 | 45 | 24 | 22 | 25 | 25 | 73 | 14 | |
| 16 | 72 | Left | 28 | 3 | 51 | | elbow flex: 3; wrist flex: 3; FDS: 3; FDP: 3 | 38 | 0 | 11 | 19 | 14 | 45 | 34 | |
| 17 | 57 | Left | 28 | 8 | 24 | | arm IR: 1; arm pronator: 1; elbow flex: 2; FDS: 2; FDP: 2; FLP: 2 | 37 | 0 | 0 | 9 | 9 | 19 | na | |
| 18 | 50 | Right | 23 | 0 | 30 | | elbow ext: 2; elbow flex: 2; wrist flex: 3; FDS: 3; FLP: 3 | 0 | 59 | 0 | 14 | 9 | 24 | 36 | |
| 19 | 82 | Left | 25 | 0 | 20 | | — | 38 | 10 | 19 | 25 | 25 | 70 | na | |
| 20 | 83 | Left | 29 | 0 | 36 | | — | 50 | 38 | 26 | 25 | 14 | 66 | 14 | |
| 21 | 62 | Left | 26 | 2 | 45 | | arm IR: 2; arm pronator: 2; elbow ext: 2; elbow flex: 1; FDS: 1; FDP:1 | 55 | 0 | 0 | 14 | 14 | 29 | 15 | |
| 22 | 55 | Left | 28 | 2 | 7 | | FDS: 1; FDP: 1 | 31 | 0 | 0 | 0 | 0 | 1 | 89 | |
| 23 | 55 | Left | 27 | 0 | 46 | | arm abductor: 2; arm IR: 2; elbow ext: 1+; elbow flex: 3; wrist ext: 3; FDS: 3; FDP: 3 | 58 | 0 | 0 | 14 | 9 | 24 | 15 | |
| 24 | 50 | Right | 14 | 6 | 10 | | inger ext: 1; FDS: 1; FDP:1; FLP: 1 | 0 | 42 | na | na | na | na | na | |
| 25 | 63 | Left | 27 | 4 | 29 | | arm IR: 1+; elbow flex: 2; wrist flex: 2; FDS: 2; FDP: 2 | 54 | 0 | 19 | 14 | 14 | 14 | 47 | |
| 26 | 59 | Left | 26 | 3 | na | | — | 45 | 0 | 0 | 0 | 0 | 1 | na | |
| 27 | 77 | Left | 25 | 9 | 37 | | — | 29 | 26 | 26 | 33 | 33 | 93 | 18 | |
| 28 | 56 | Right | 15 | 4 | 9 | | arm pronator: 1; elbow flex: 1; wrist flex: 1; FDS: 1 | 0 | 44 | 22 | 14 | 14 | 51 | na | |
| 29 | 42 | Left | 26 | 0 | 4 | | — | 25 | 12 | 26 | 25 | 25 | 77 | na | |
| 30 | 62 | Right | 26 | 0 | 19 | | — | 0 | 43 | na | na | na | na | na | |
| 31 | 71 | Left | 28 | 0 | 17 | | arm pronator: 1; elbow flex: 1 | 40 | 1 | 11 | 14 | 14 | 40 | na | |
| 32 | 67 | Left | 27 | 0 | 51 | | — | 66 |  |  |  |  |  |  | |
