# Supplementary material for: Feasibility and User Experience of Immersive Virtual Reality–Based Rehabilitation in Patients With Stroke: Single-Arm Pretest-Posttest Pilot Study
Source: JMIR Serious Games. 2026 Mar 11;14:e79584. doi: 10.2196/79584 (PMC12978540; doi:10.2196/79584)
Supplement: Checklist 1 [file games-v14-e79584-s002.pdf]

Multimedia Appendix 2. The RATE-XR Checklist as described in Vlake, J. H & RATE-XR Expert Group. (2024). Reporting Guidelines for the Early-Phase Clinical Evaluation of Applications Using Extended Reality: RATE-XR Qualitative Study Guideline. *Journal of medical Internet research*, *26*, e56790.

Theme	Item number	Recommendation	Page number(s)	Note(s)/Quote(s)
Title and abstract				
Title	1	Identify the study as an early clinical evaluation, or a similar term, of an application using XR[b], or a more specific term, in the title, including its intended aim.	1	Feasibility and User Experience during Immersive Virtual Reality-based Rehabilitation in Patients with Stroke: Single-Arm Pretest-Posttest Pilot Study
Abstract	I	Provide a (structured) summary of the study.<br><br>Consider including the following:<br><br>1) A concise description of the clinical problem or knowledge gap and the rationale for using an application using XR<br><br>2) A concise description of the study methods, including a short description of the application including its name, study population, study setting, main outcomes, and assessment methods.<br><br>3) A concise description of the results, including safety and harm outcomes<br><br>4) A short conclusion<br><br>5) If applicable, details about the registration of the study in a publicly available database.	1	(1) "*However, existing work often lacks a structured assessment of user experience over a longer period of time"*<br><br>(2) "*Thirty-two chronic (N=19) or post-acute (N=13) patients aged 60±11 years with stroke were enrolled. They all performed 4 weeks of training, performing exercises in the Virtual Supermarket for Stroke (VSS)."*<br>[…]<br>*"We assessed subjective outcomes after the first and the last session. Clinical scales were administered at the baseline and at the end of the treatment*."<br><br>(3) see Abstract subsection Results.<br><br>(4) see Abstract's Conclusions.<br><br>(5) n.a.
Introduction				
Clinical problem and existing evidence	2	Introduce the clinical problem for which the application using XR was used, including its relevance and a description of (the efficacy of) evidence-based or commonly used	2	*"Current literature suggests that positive outcomes can be achieved through functional training and pharmacological treatments"*

		interventions or the treatment as usual, which is intended to be replaced by the application using XR.		*"Although precise guidelines have yet to be defined, it is currently agreed that adherence to the proposed intervention is essential. The importance of continuing to exercise has been emphasized in several studies, which have highlighted the potential of intensive, task-specific, and meaningful exercises to recover sensory motor functions [11, 12]"*
Introduction of the application	3	Introduce the application using XR, including the following:<br><br>(1) Hypotheses for the potential effect; how the application is expected to contribute to the clinical problem.<br><br>(2) If available, a concise description of, or a reference to, previous research on the same (or a similar) application.	3,4	(1) "*an immersive VR application specifically developed to support cognitive functions and upper limb rehabilitation"*<br><br>(2) "*The immersive VR application object of this work is derived from previous work carried out by our research group in previous years. In particular, we started with the development and validation of a virtual supermarket dedicated to the training of visuo-spatial abilities in which people could walk naturally; we assessed and demonstrated its usability and acceptance in a group of healthy young adults [31], and in older adults with mild cognitive decline or subjective cognitive decline [32]. Later, we started developing a second version with a simplified interaction, which was tested in the healthy population with positive results [33]."*
Objectives	II	Specify the study objectives or hypotheses.	4	"*We hypothesized that the chance of experiencing an ADL with a customized and adaptable level of difficulty could help maintain the flow levels of the exercise, as well as have other positive effects, and positively impact the sense of presence [22] and emotional state. Moreover, we expected the intervention to be feasible, with patients being able to interact with the application and not experiencing any adverse symptoms due to cybersickness.*"
Methods and analysis				
Trial design and reporting	III	Provide a reference to ethical approval and, if available, to any (published) study protocol and registration of the study in a publicly available repository.	4, sect. Ethical Considerations	"*The trial protocol has been approved by the Insubria Ethical Committee (ref. no. 70, 01/12/2020)"*
Trial design and reporting	IV	Describe, and mention the rationale for, the study design. For clarification, it is recommended to use a flow diagram.	4, sect. Methods	*"The study was an interventional single-arm pretest-posttest study that aimed at evaluating the feasibility, the user experience, and the preliminary impact of VSS 3 times a week for 4 weeks in a sample of participants with stroke"*

Participants and setting	4	Describe the setting and locations, including country, where data were collected and processed, and where the application using XR was applied and evaluated.	4, sect. Participants	*"[..] Villa Beretta rehabilitation center (Costa Masnaga, LC, Italy), the rehabilitation department of Valduce Hospital, where the study was carried out."*
Participants and setting	5a	Describe how participants were selected and recruited and provide eligibility criteria.	4,5, sect. Participants	"Participants were recruited through convenience sampling among patients with stroke"<br><br>*"Participants were either in the post-acute (time from the stroke longer than 15 days) or chronic phase (more than 6 months). They all met the following inclusion criteria: age above 18 years; clinical stability; absence of pain, postural instability, muscle hyperactivity, and impairments that prevent the accomplishment of the reaching task; Mini-Mental State Examination (MMSE) 20 or an equivalent cognitive level for patients with aphasia. Exclusion criteria were a history of seizure or motion sickness, severe visual deficits, and inability to provide informed consent."*
Participants nad setting	5b	Describe who will be applying the application and whether they were trained.	6, sect. Equipment	*"All the therapists/psychologists involved in the study were trained by the VSS developer to set up the game area and use the application, which was developed by the researchers who created the VSS."*
Intervention and procedures	6	Provide a description of the application, including its content, hardware, protocol, and set-up, or provide a reference to previous publications where this information is described. Consider supplementing the description with an image, figure, or film.	5, 6, sections Equipment and Protocol	"*The VSS is an immersive VR application [..]. It was developed with Unity and deployed for Oculus Rift v2.*"<br><br>"*The VR environment is constituted by two scenarios. The first is devoted to picking groceries from the shelf (Figure 1); the second is paying for such items (Figure 2)."*
Intervention and procedures	7	Describe, or provide a reference to, the development process of the application.	5, sect. Equipment	*"It was developed with Unity and deployed for Oculus Rift v2.*"
Intervention and procedures	8	Describe the participant timeline in sufficient detail to allow replication, including all procedures, co-interventions (if applicable), and (follow-up) assessments.	6, sect. Protocol<br><br>7, Sect. Outcomes	*"Each patient underwent 12 sessions of 20 minutes, in which the tasks of doing the shopping and paying at the cash register were repeated recursively. The therapist chose the level of difficulty, the presence of distractors, and other elements that increase the complexity of the task, both at the beginning and during the session. All sessions occurred in a quiet room, where only the patient and the therapist were present."*

				All outcome measures are reported and described in sect. Methods > Outcomes.
Intervention and procedures	V	Describe and give a rationale for the control conditions or provide a rationale for not using one.	n.a.	See study design.
Outcomes	VI	Describe all prespecified primary and secondary outcomes, including how and when assessed.	7, Sect. Outcomes	All outcome measures are reported and described in sect. Methods > Outcomes.
Outcomes	9	Describe how safety and harm outcomes were assessed. Describe which, and how, other XR-specific outcomes were assessed, such as performance, usability, presence, perspectives, and acceptability.	7, Sect. Outcomes	XR-related measures:<br>(i) the Short Flow Scale (SFS);<br>(ii) the International Test Commission - Sense of Presence Inventory (ITC-SOPI);<br>(iii) the Positive and Negative Affect Schedule - Short Form (I-PANAS-SF);<br>(iv) the Simulator Sickness Questionnaire (SSQ);<br>(v) the Technology Acceptance Model (TAM3) subscale for the evaluation of perceived ease-of-use (PEOU)
Sample size	VII	Provide a justification for the sample size.	6, sect. Participants	"*The sample size was calculated using the formula reported in Candel and van Breukelen for single-arm pre-post studies [34], and considering flow (specifically short flow scale – see sect. Outcomes) as main outcome.*<br>*[..]*<br>*we computed a sample of 23 participants using SD=1.2, alpha = 0.05, power = 80%, and expecting a pre-post variation equal to 0.65. We then rounded the final sample size to 30, considering the possibility of 20% drop-outs and aligning the final sample to other studies assessing feasibility [36].* "
Analysis	VIII	Provide a detailed description of how primary and secondary outcomes were analyzed, including any prespecified comparisons or stratifications.	8, 9, sect. Stastical Analysis	All data analyses are reported in sect. Methods > Statistical Analysis
Protocol alterations	IX	Describe changes to the methods or protocol, including procedures, study outcomes, eligibility criteria, and analysis plan, after study commencement, with	n.a.	n.a.

		reasons, and, if applicable, report whether the study registration was updated.		
Results				
Participant flow and recruitment	X	Describe the time frame of recruitment and follow-up and the participant flow, including the number of patients screened and included, receiving the intervention, and being included in each analysis. Report if, and why, the study was prematurely terminated. The use of a flow diagram is highly recommended.	9, sect. Results	*"All patients except one completed the training; one participant left the study because they did not feel familiar with and did not feel at ease while interacting with technology"*
Baseline data	XI	Describe, or add a table depicting, baseline and treatment-related characteristics. If applicable, describe and specify any concurrent measures.	9, Supplementary material	*"Demographic and baseline data of the study participants are presented in Table 1 of Supplementary Material."*
Main results	XII	Report on all prespecified outcomes that are available. Consider using tables, figures, or graphs to illustrate results.	9, 10, 11, 12; Sect. Results > User experience and > Clinical Outcomes	We had data reported in Table 1 and Table 2; Figure 4 and Figure 5.
XR and human factors	10	Include information about the usage of the application, such as duration, frequency, number of sessions, error rates, and number of sessions requiring interruption or discontinuation, including reasons.	6, sect. Protocol	"*Each patient underwent 12 sessions of 20 minutes, in which the tasks of doing the shopping and paying at the cash register were repeated recursively."*
XR and human factors	11	If assessed, report on XR-specific outcomes, such as performance, usability, presence, perspectives, and acceptability.	9, 10, Sect. User Experience	See Sect. User Experience
Safety and harms	12	Report on safety and harms, including unintended effects, both during and after using the application.	9, 10, Sect. User Experience	*"no adverse events were recorded"*<br><br>We also report results of cybersickness assessment in Table 1.
Participant flow and recruitment	X	Describe the time frame of recruitment and follow-up and the participant flow, including the number of patients screened and included, receiving the intervention, and being included in each analysis. Report if,	4, sect. Methods<br><br>9, sect. User Experience	We only report that: "The study was carried out between April 2023 and February 2024."<br><br>"*One participant left the study because they did not feel familiar with and did not feel at ease while interacting with technology.*

		and why, the study was prematurely terminated. The use of a flow diagram is highly recommended.		*For this participant, only clinical scales were collected at T1, leading to user experience-related results being calculated on N=31. There were no other missing data.*<br>*Missing data in the clinical scales were evaluated by recoding non-participation cases (e.g., patients unable to walk could not perform the Time Up and Go test at any time point) as valid scores. Little's MCAR test was not significant (Chi2 = 69.201, p = 0.72), indicating that other missing data were random*."
Discussion and conclusion				
Generalizability and impact	13	Discuss (potential) impact of study findings and generalizability, including barriers for the use and implementation of the application.	13	*"The designed intervention was feasible, with excellent adherence among stroke survivors (97%). The general experience was positive, and participants welcomed the possibility of rehabilitation with the support of immersive technologies, which also led to some improvements in functional outcomes."*
Safety and harms	14	Discuss safety and instances of harm, including their possible effects on study findings, implications for future use of the applications, and whether they can be prevented or mitigated.	13, 14 sect. Discussions<br><br>16, sect. Conclusions	*"The observed improvements in upper limb dexterity (Box-and-Block Test) and motor strength (Motricity Index) suggest that the intervention may translate into meaningful functional gains in daily activities such as reaching, grasping, and manipulating objects [63]. The enhanced balance and mobility indicate potential benefits for overall independence and fall prevention [64]."*<br><br>*"From a clinical perspective, the intervention appears feasible and well-tolerated"*
Ethics	15	Describe ethical considerations, including benefits and risks, for the current and future use of the application.	(partially – no ethical considerations)<br><br>15, sect. Discussions > Clinical outcomes<br><br>16, sect. Conclusions	"*Its simple setup and ease of use make it potentially adaptable to a variety of clinical settings, and potentially for future use at home or in unsupervised contexts (e.g., with a stand-alone head-mounted display and automatic progression of difficulty). In the future, it would be valuable to increase the difficulty levels and include different interaction technologies (e.g., hand tracking) to make the application more accessible to patients with limited arm motor function, thereby providing a longer training period."*
Strengths and limitations	XIII	Discuss study strengths and limitations, including sources of potential bias.	15,16, sect. Limitations	*"First, the sample size was small and estimated based on the assessment of our main outcome, i.e., flow; thus, the statistical power may not be sufficient to draw conclusions at the clinical*

				level. Second, the population was heterogeneous, including both patients in the sub-acute and chronic phases who completed the exercise with either the impaired or less-impaired side. Finally, we did not have a control group."
Conclusion	16	Provide a conclusion that accurately interprets study findings, including future perspectives.	16	See sect. Conclusions
Generalizability and impact	13	Discuss (potential) impact of study findings and generalizability, including barriers for the use and implementation of the application.	16	See sect. Conclusions
Statements				
Funding and conflicts of interest	XIV	Disclose any potential conflict of interest, real or apparent, including the funding sources and their roles in the design, conduct, analysis, and report of the study, potential roles of commercial companies, and personal conflicts of interest for each author.	16	See sect. Conflicts of Interest
Application	17	Indicate whether the application is a commercial product, it is publicly available, it can be accessed, it complies with the medical device regulations, and whether the application was approved for its intended use by a formal regulatory body or if the study is part of the clinical evaluation for future certification.	(partially addressed)<br><br>3, Sect. Introduction	"*an immersive VR application specifically developed to support cognitive functions and upper limb rehabilitation. The decision to develop an ad-hoc application was made to avoid the limitations commonly associated with commercial games, including inaccessible interactions for people with disabilities, a lack of ecological validity, and difficulty in customization*"
